# Oscillatory electrostatic potential on graphene induced by group IV element decoration

**DOI:** 10.1038/s41598-017-13603-w

**Published:** 2017-10-13

**Authors:** Chunyan Du, Liwei Yu, Xiaojie Liu, Lili Liu, Cai-Zhuang Wang

**Affiliations:** 1 Center for Quantum Sciences and School of Physics, Northeast Normal University, Changchun, 130117 China; 2 Department of Chemistry, School of Science, Beijing Technology and Business University, Beijing, 10084 China; 3 Ames Laboratory – US Department of Energy, and Department of Physics and Astronomy, Iowa State University, Ames, IA 50011 USA

## Abstract

The structures and electronic properties of partial C, Si and Ge decorated graphene were investigated by first-principles calculations. The calculations show that the interaction between graphene and the decoration patches is weak and the semiconductor patches act as agents for weak electron doping without much disturbing graphene electronic π-bands. Redistribution of electrons due to the partial decoration causes the electrostatic potential lower in the decorated graphene areas, thus induced an electric field across the boundary between the decorated and non-decorated domains. Such an alternating electric field can change normal stochastic adatom diffusion to biased diffusion, leading to selective mass transport.

## Introduction

Graphene has been a material of current intensive studies due to its novel electronic and structural properties which make graphene an appealing system for both fundamental studies and carbon-based electronic device applications^1–4^. Synthesis of high quality graphene on a large scale is the foundation for its promising applications. Two main growth technologies have been developed aiming at achieving this goal, chemical vapor deposition (CVD) on metal substrates^5–7^ or epitaxial thermal growth on semiconductor substrates^8,9^. While graphene sample can be grown on metal substrates, it is of challenge to transfer it onto target insulator or semiconductor substrates for device fabrication. More importantly, the electronic structure of pristine graphene can be significantly affected if the graphene is supported by transition metals. The use of SiC as substrate avoids hazardous transfer of the graphene to the target substrate, but partially covalently bonded to the Si-terminated SiC substrate may also cause distortions to electronic structure of graphene^8,9^.

To overcome this limitation for applications, various methods have been investigated to decouple graphene from its substrate and to form a quasi-free-standing layer of graphene. Some species, including metals^10–12^ and non-metals^13–15^, have been successfully intercalated between the epitaxial graphene and its substrates and made graphene a quasi-free-standing layer. For example, it has been shown that the performance of graphene has been enhanced by intercalation of H between graphene and its substrates^4^.

Since the presence of metallic states underneath graphene would be disadvantageous considering perspective applications of graphene in electronic devices, intercalation of non-metallic atomic layers, such as C, Si and Ge, would be preferable^16–20^. These semiconductor materials can act as an insulating layer between graphene and underlying metal substrates. Very recently, experiments have shown that thermal treatment of epitaxial graphene with Ge detaches the buffer layer from the substrate, giving rise to a quasi-free-standing structure with two distinct stable phases: an n- and a p-doped graphene, distinguished by different concentrations of the intercalating elements^18,19^. Another semiconductor element, silicon, was also introduced as the interfacial layer between graphene and metallic Ru(0001) substrate^20,21^ which effectively weakens the interaction between graphene and ruthenium. Therefore, semiconductor buffer layers play a particularly important role in modifying interfaces between graphene and underlying substrates.

In fact, in experiments, the graphene is partially decorated with either Si or Ge. For example, Baringhaus *et al*.^18,19^ prepare both p- and n-type doped graphene areas on SiC(0001) substrate by non-homogenous Ge intercalation. In their samples, both residual pristine graphene areas and non-homogenous Ge intercalation are coexisted. Similarly, Si patches have been intercalated into the interface between graphene and SiC or Ru substrates^16,19,20^, leading to complicated phases both with Gra/SiC areas and Gra/Si/SiC areas. Therefore, it is of great interest to investigate the properties of graphene partially intercalated by a non-metal layer. So far our knowledge about such interactions and the consequent emerging properties are still limited.

In this study, we model partially C, Si, Ge, Si-Ge intercalated graphene by first-principles calculations. Since the calculations for partially intercalation with the presence of substrate will require much large number of atoms than current first-principles methods can handle, we simplify the calculations by neglecting the substrate. In such a setup, the partially C, Si, Ge, Si-Ge intercalated graphene can be view as partially C, Si, Ge, Si-Ge decorated graphene. Therefore, we will use the terminology of partially decorated graphene to represent partially intercalated graphene in the rest of the paper. Although the experimental structures mentioned above are obtained by nonmetal intercalation, such structures can also be considered as semiconductor decoration from chemical view. We show that the interaction between graphene and C, Si, Ge, or Si-Ge layer is of Van der Waals character, which retains the graphene electronic π-bands. The decorated C, Si, Ge, or Si-Ge layers act as agents for electron doping of graphene. The redistribution of the electrons makes the electrostatic potential different across the domain between the decorated and non-decorated areas or domains of different decorated elements therefore induce alternating electric field. Such an alternating electric field would change normal stochastic adatom diffusion to biased diffusion, leading to selective mass transport^10,22^ and consequent nucleation on either the decorated or pristine areas. The effect would be utilized to control self-organized nanostructures on graphene at the atomic level^10,11^.

## Computational Methods

**Figure 1:**
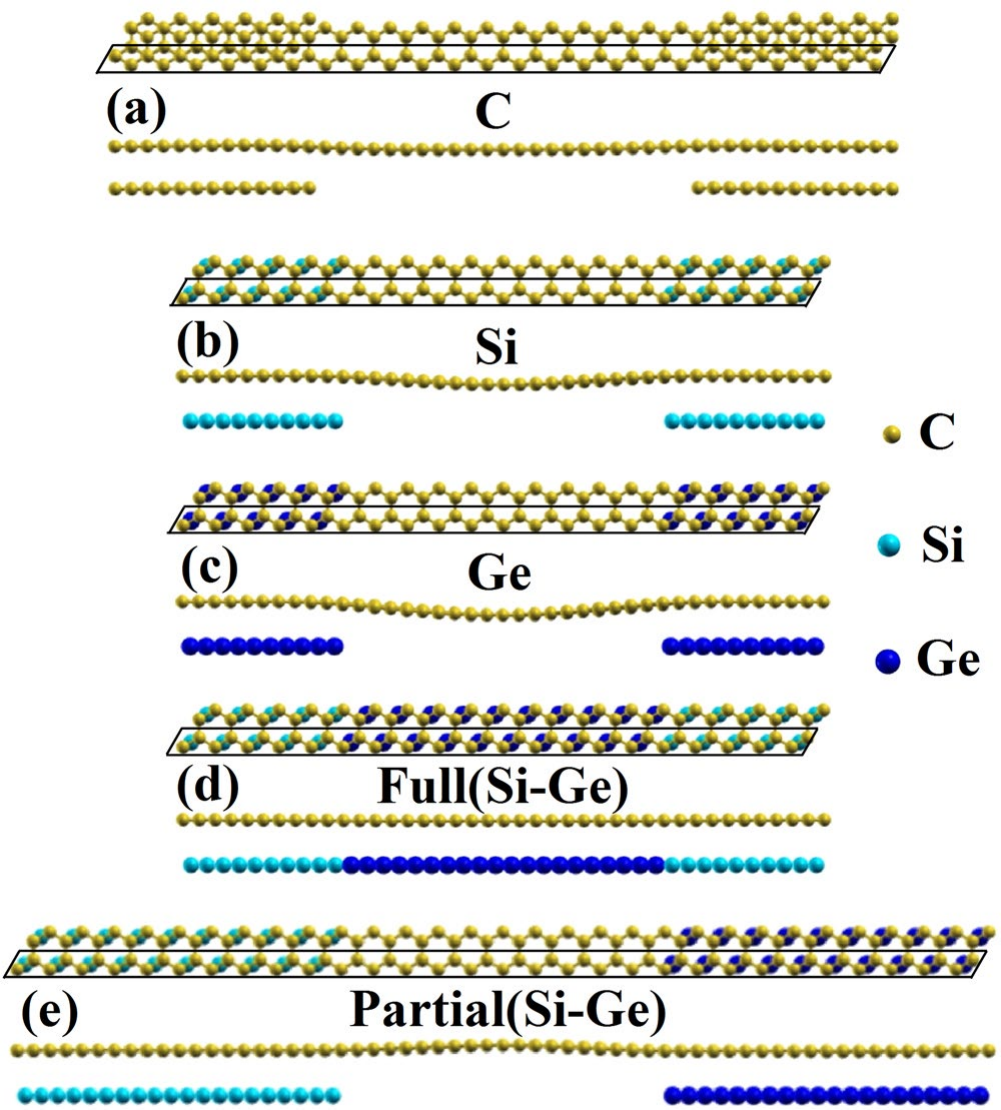
Geometries of partially C (**a**), Si (**b**), Ge (**c**), fully Si-Ge (**d**), as well as partially Si-Ge (**e**) decorated graphene. Gold balls indicate carbon atoms; cyan balls present silicon atoms, and blue balls shows germanium atoms. The black boxes present the supercell in the calculations.

The atomic structures used in the calculations contain both decorated and pristine areas as shown in Fig. 1(a)~(e). For C partially decorated graphene, a 24 × 1 × 1 graphene supercell is used and a 6 × 1 graphene patch is attached underneath each end of the graphene supercell as shown in Fig. 1(a). A 20 × 1 × 1 graphene supercell is used to model partially Si or Ge decorated graphene, with a 5 × 1 Si or Ge patch underneath each end of the graphene supercell as can be seen from Fig. 1(b) and (c). We also model a 20 × 1 × 1 supercell of graphene with a underneath 10 × 1 Si and a 10 × 1 Ge patches as a full Si-Ge co-decorated graphene as shown in Fig. 1(d). Finally, a model with a 30 × 1 × 1 supercell of graphene with a 10 × 1 Si decorated layer on one end, a 10 × 1 Ge decorated layer on the other end, and a 10 × 1 pristine graphene in the middle respectively, as shown in Fig. 1(e), is also studied. In the structure models discussed above, the dimension of supercell used in the calculation along **a** and **b** directions are 59.04 Å and 2.46 Å for the partially C decorated graphene, 49.2 Å and 2.46 Å, respectively, for the partial Si, Ge decorated graphene. The values of **a** and **b** are 73.8 Å and 2.46 Å for partial Si-Ge co-decorated graphene, and 49.2 Å and 2.46 Å for full Si-Ge co-decorated graphene. The dimension along **c** is more than 17.5 Å in all models, which allows at least 14 Å of vacuum to separate the two surfaces. Periodic boundary conditions are applied. To mimic the partial intercalation experiments, the edges of the decorated C, Si, and Ge patches are not passivated. A Gaussian smearing with a width of σ = 0.05 eV are used in the calculations. All atoms in the supercell are allowed to relax until the forces on each atom are less than 0.01 eV/Å. The supercell dimension and the decorated layers are kept fixed during the relaxation.

The first-principle calculations are performed based on the density functional theory (DFT) with generalized gradient approximation (GGA) for the exchange-correlation energy functional in the form of Perdew-Burke-Ernzerhof (PBE) functional^23,24^ implemented in the VASP^25–27^ code, including dipole moment corrections^28^. The valence electrons are treated explicitly and their interaction with ionic cores are described by projector augmented wave pseudopotential^29,30^. Van der Waals interactions by the D2 method of Grimme^31^ have been carried out to describe the long range dispersion. For C, Si and Ge atoms, the 2 s^2^ and 2p^2^ electrons are treated as valence electrons. The wave functions are expanded in a plane wave basis set with an energy cutoff of 400 eV. A k-point sampling of 1 × 20 × 1, 1 × 24 × 1, 1 × 10 × 1 Monkhorst-Pack grids respectively in the first Brillouin zone are used for partial Si, Ge and full Si-Ge co-decorated graphene, partial C decorated graphene, and partial Si-Ge co-decorated graphene.

In principle, substrate must be considered to model the experimental situations^32,33^ because the structure of the intercalation can be affected by the substrate if the interaction between the intercalant and substrate is strong. However, for dealing with partial intercalation, the number of atoms in the supercell would easily exceed 1000 if several layers of substrate are included^33^. This is because the likely lattice mismatch between the intercalation layer and the substrate will require not only a large dimension along the ***a*** direction to accommodate the partial intercalation, but also a large supercell along the ***b*** direction to accommodate the lattice mismatch. Without substrate we can use a small ***b*** vectors as the model used in present calculations so that the number of atoms can be manageable with our available computational resource. In some cases, the structure of intercalation layer can be determined by experiment^10,11^. In such cases, DFT calculation can be carried out by adopting the structure model suggested by experiment. In order to get some estimation on how big effects on the properties of the intercalated graphene will be by adopting the experimental structure model for the intercalation layer but neglecting the substrate, we calculate the absorption energy of a sodium adatom on graphene decorated with a full Si layer with and without a graphite substrate. The calculation results show that the adsorption energy for Na on graphene by full Si decoration with and without graphite substrate is −0.95 eV and −1.10 eV, respectively. These results suggest that while the substrate does have some effects on the absorption properties of Na adatom on graphene surface, but such effects is not large as long as the interaction between the substrate and the intercalation layer is not larger.

## Results and Discussions

Three types of geometries are used to study the properties of semiconductor decorated graphene: 1) partially decorated graphene with only one type of element as shown in Fig. 1(a)~(c); 2) fully decorated graphene with two different elements, one on each half of the graphene as shown in Fig. 1(d); 3) partially decorated graphene with two different elements, one on each end of the graphene as shown in Fig. 1(e). All structures shown in Fig. 1 are after the relaxation by first-principles calculations. Significant buckling is found for all the partially decorated graphene, while the geometries of fully decorated graphene are essentially flat with negligible vertical distortions. The calculation results show that the interlayer distances are 3.34, 3.51, 3.45, 3.48, and 3.85 Å, respectively, for partially C, Si, Ge, fully Si-Ge, and partially Si-Ge co-decorated graphene. These results indicate that the interaction between graphene and C, Si, Ge, and Si-Ge patches is weak and of Van der Waals character, similar to the case of interlayer interaction in graphite.

**Figure 2:**
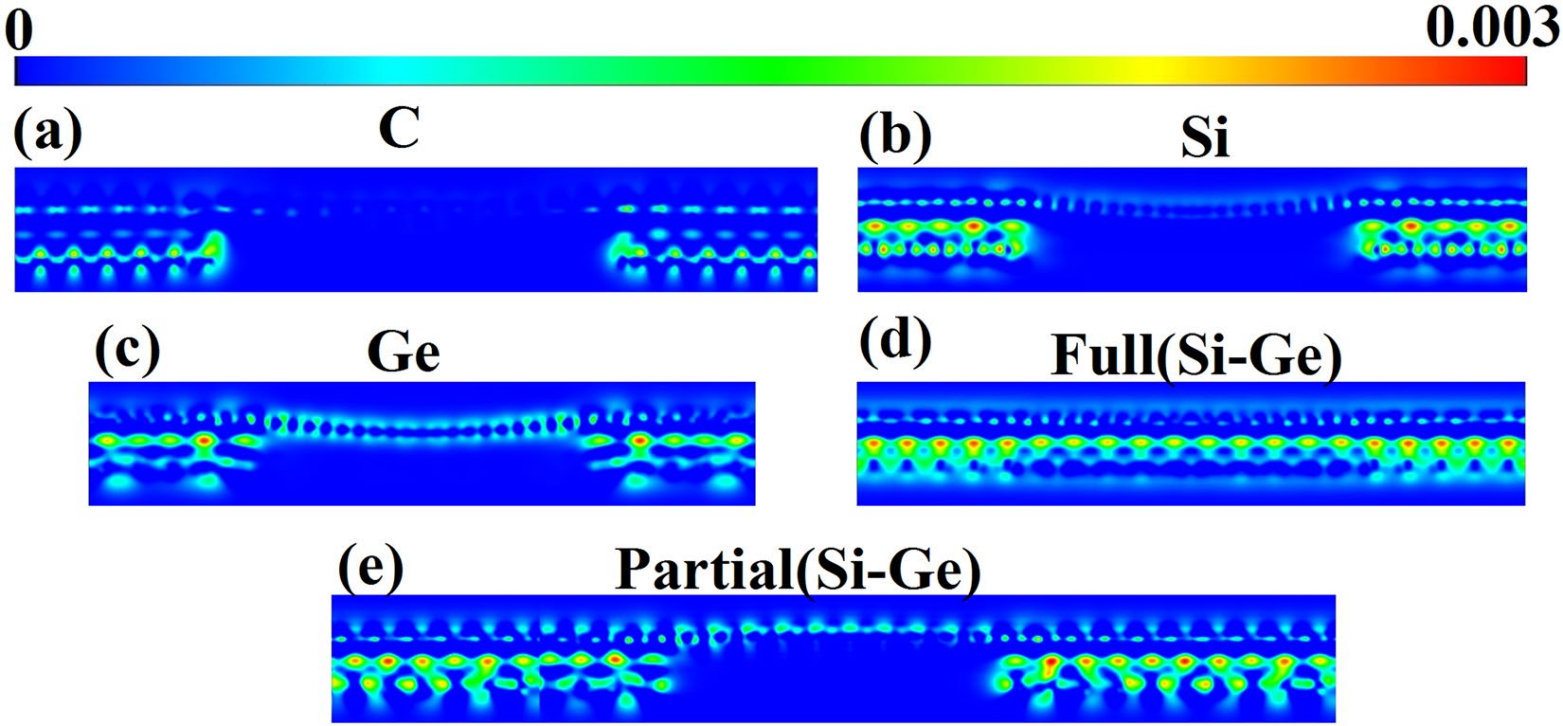
Interaction charge density (ICD) distributions (in electron/Å^3^) induced by partial C (**a**), Si (**b**), Ge (**c**), full Si-Ge (**d**), and partial Si-Ge (**e**) decoration. ICD is defined as Δρ(*r*) = ρ(*r*) − ρ_gra_ (*r*) − ρ_patch_(*r*). The interaction charge density shown in color map is a relatively value and given in unit of electron/Å^3^.

First, we calculate the interaction charge density (ICD) distributions to gain the insight about the interaction between the graphene and the decorated semiconductor patches. The interaction charge density distributions Δρ(r) is defined as Δρ(r) = ρ(r) − ρ_gra_(r) − ρ_patch_(r), where ρ(r) is the charge density of whole system, and ρ_gra_(r) and ρ_patch_(r) are the charge densities of ideal graphene and the underlying decorated patch, respectively, calculated using the same supercell setups. The results are shown in Fig. 2. Positive values (red) in Fig. 2 indicate increases in the electron density after C, Si, Ge, and Si-Ge decoration, while negative values (blue) indicate electron density reductions. The interaction charge density defined in this way accounts for the electron redistribution due to the interaction between the graphene and the underlying decorated patches. From Fig. 2 we can see that the charge density is mainly located at interface between graphene and the underlying decorated patches. Although the interaction between graphene and decorated patches is of Van der Waals nature as discussed above, charge transfer calculated based on Bader analysis^34^ shows that there are charge transfers from the C, Si, Ge, and Si-Ge patches to graphene as listed in Table 1. It should be noted that the value of the electron transfer from underlying decorated patches to graphene is larger for Ge (i.e., 0.19), followed by Si (i.e., 0.08), and then C (i.e., 0.02) as can also be seen from Fig. 2. These results indicate that the interaction between Ge patch and graphene is slightly stronger than that of Si and C patches. Charge transfer is much more complicated in the case of fully and partially Si-Ge co-decorated graphene. The total charge transfer of partially Si-Ge decorated graphene is 0.31 electrons and larger than that of fully Si-Ge co-decorated graphene which is 0.23 electrons. Thus the decorated patches donate electrons to graphene layer, making graphene slightly n-doped.

**Table 1:** Charge transfer calculated by Bader analysis and integrated density of state (DOS) (charge transfer indicated by superscript a) from Dirac point to the Fermi level. Note that positive signs indicate gain electrons, and negative signs indicate loss electrons.

Δq (e)	Partial C	Partial Si	Partial Ge	Full Si-Ge	Partial Si-Ge
Gra	+0.02	+0.08	+0.19	+0.23	+0.31
C	−0.02	—	—	—	—
Si	—	−0.08	—	−0.12	−0.12
Ge	—	—	−0.19	−0.10	−0.19
Gra	—	+0.06^a^	+0.09^a^	+0.21^a^	+0.29^a^

**Figure 3:**
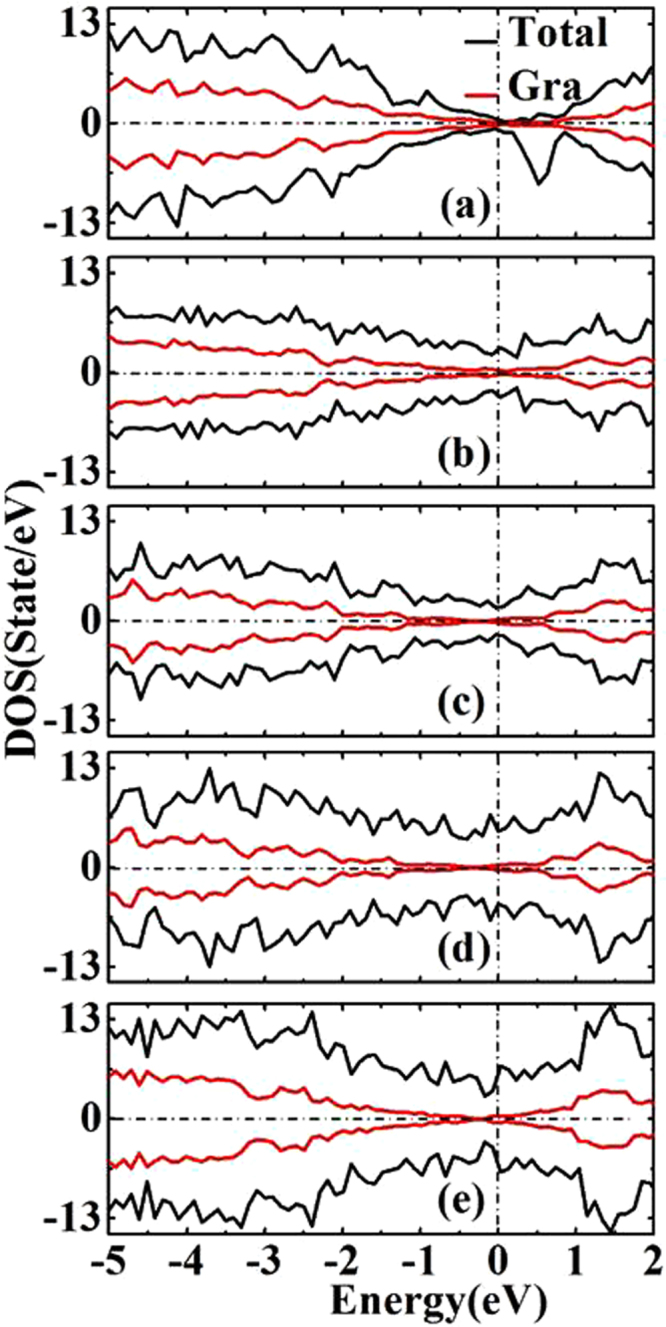
The total (black lines) and local (red lines) electronic density of state (DOS) of partially C (**a**), Si (**b**), Ge (**c**), fully Si-Ge (**d**), and partially Si-Ge (**e**) decorated graphene. The Fermi energy is shifted to zero energy as indicated by the vertical dash line. The two spin polarizations are shown above and below the horizontal axis.

In order to see more clearly the effects of the decoration on the electronic structures of graphene, we have studied the spin-polarized DOS by three times of K-point sampling used in geometry relaxations. Figure 3 shows the total DOS (black lines) of decorated graphene, and partial DOS (red lines) of the top graphene layer in the neighborhood of the Fermi level (E_F_ = 0 eV). The DOS for partially C, Si, Ge, fully Si-Ge, and partially Si-Ge co-decorated graphene are very similar. The Dirac point and the DOS of the graphene below the Dirac point are well preserved upon the C, Si, Ge, and Si-Ge co-decoration, but the Fermi level shift up by 0.08, 0.16, 0.18, 0.35 and 0.55 eV respectively away from the Dirac point. These results are consistent with the electron transfer analysis discussed above and also show that electron transfer from the underlying decorated patches to graphene which makes graphene slightly n-doped. By examining the Fermi level shift with respect to the Dirac point, charge transfer can be calculated as shown in Table 1. The value of charge transfer is similar to that of from Bader analysis.

**Figure 4:**
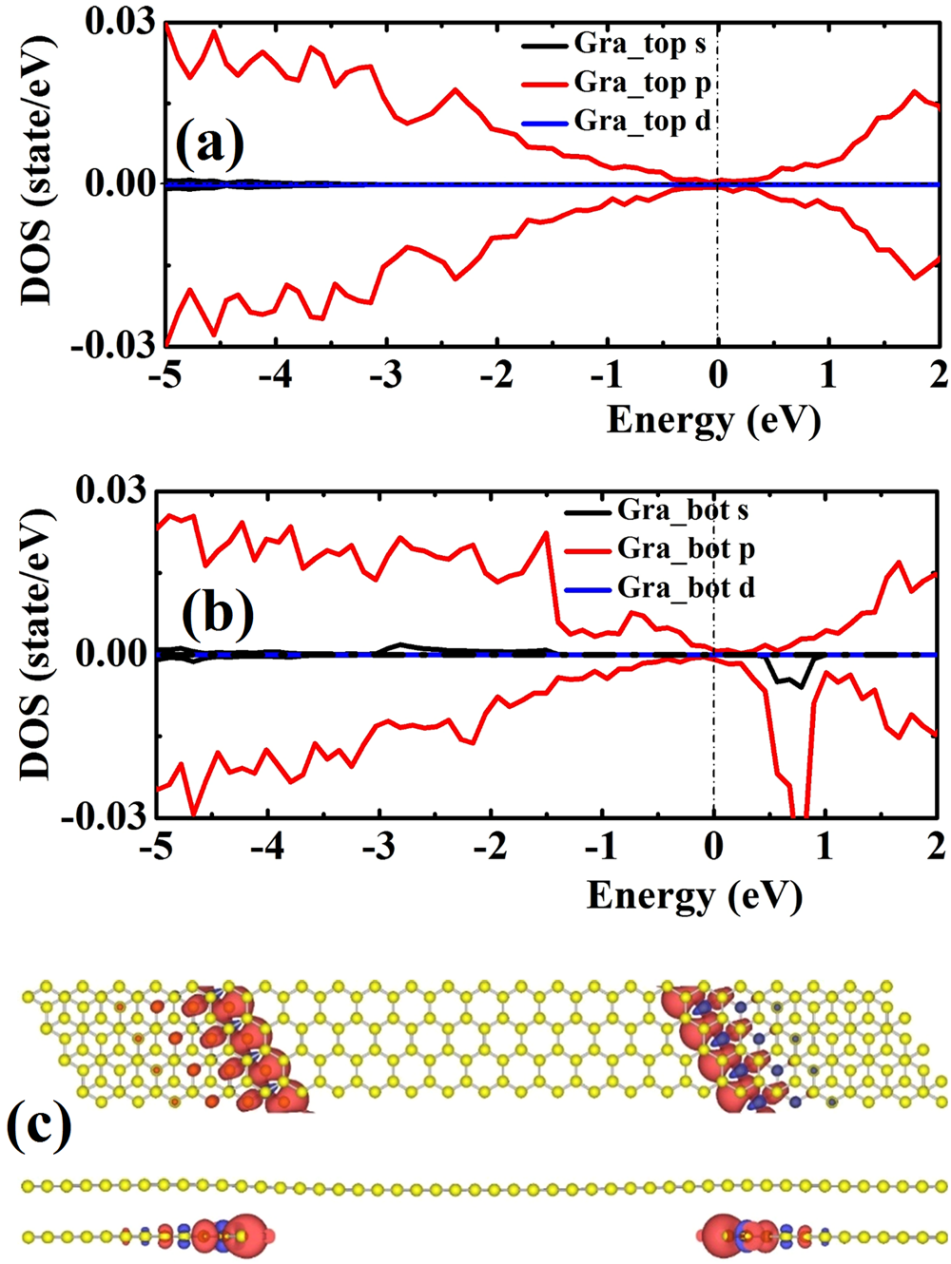
(**a**)~(**b**) The normalized local DOS of partially C decorated graphene. The Fermi energy is shifted to zero energy as indicated by the vertical dash line. (**c**) Spin charge density of partially C decorated graphene. The charge density isosurfaces is 0.015 electron/Å^3^. Blue color indicates spin-down charge density and red color indicates spin-up charge density.

In the case of Si, Ge, and Si-Ge co-decorated graphene as shown in Fig. 3(b)~(e), the spin-up and spin-down density-of-states (DOS) exhibit symmetrical distribution and induce no magnetic moments. However, for partially C decorated graphene as shown in Fig. 3(a), the occupied spin-up and spin-down states are uneven, indicating some magnetic moments in the system. In order to see the origin of magnetic moment in partially C decorated case, we plot DOS of top and bottom carbon layer separately as shown in Fig. 4. For the DOS of top carbon (i.e., the graphene) layer, the spin-up and spin-down states are almost symmetric, leading to almost zero magnetic moments as one can see from Fig. 4(a). However, the DOS of the underlying decorated carbon patches as shown in Fig. 4(b) is unsymmetrical, resulting in some magnetic moment in the system. The calculation result shows that the magnetic moment is mainly from the underlying decorated carbon patches, especially from the zigzag edge^35^ due to dangling bonds at the edge of the underlying decorated carbon patches. This result is also confirmed by the spin charge density defined as Δρ(r) = ρ_up_(r) − ρ_dw_(r), where ρ_up_(r) and ρ_dw_(r) are the electron densities of spin-up and spin-down separately, as shown in Fig. 4(c).

**Figure 5:**
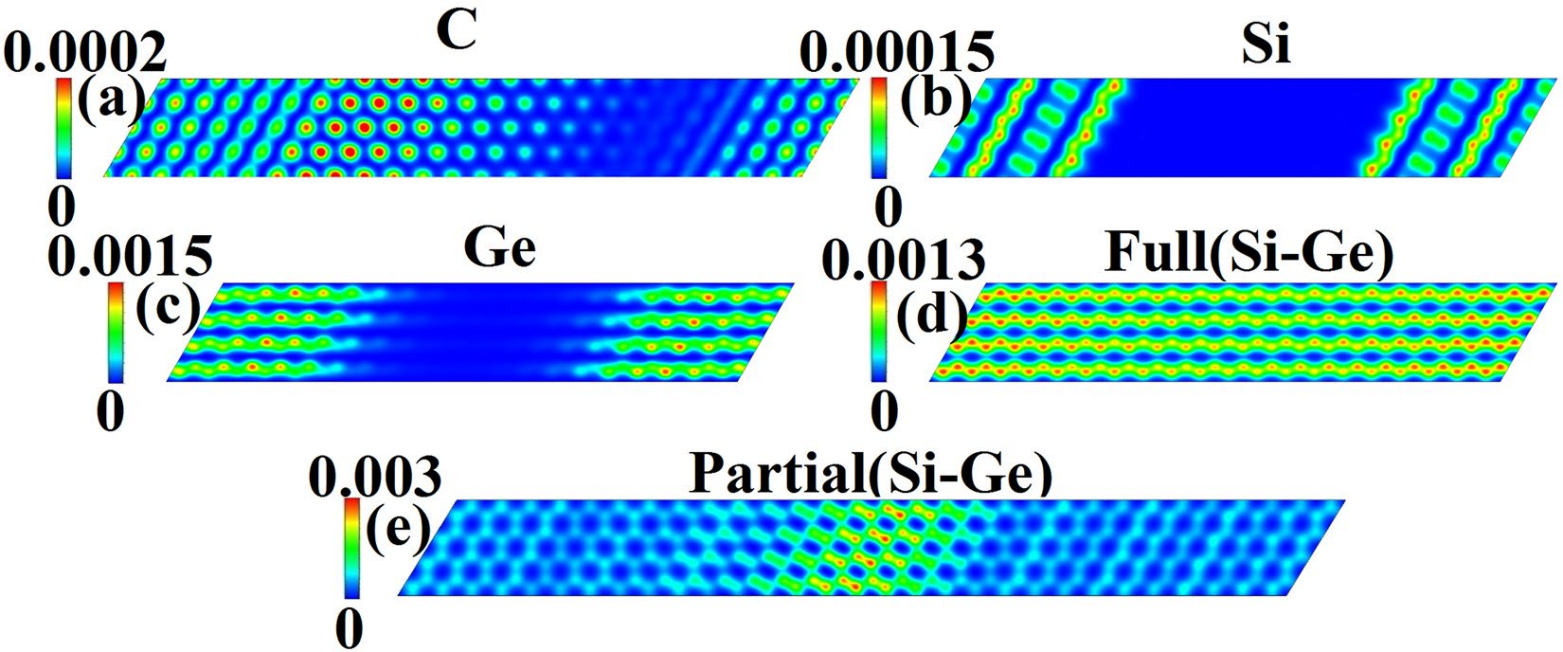
Partial charge density distribution of partially Si (**a**), Ge (**b**), fully Si-Ge (**c**), partially Si-Ge (**d**) decorated graphene, in the energy window from Dirac point to the Fermi level on the horizontal plane cutting at 1 Å above graphene layer.

As discussed above, the interaction between graphene and the underlying semiconductor decorated patches is weak and the decorated semiconductor patches act as agents for weak electron doping without much disturbing on the graphene electronic π-bands. Due to electron transfer from the underlying decorated patches to the graphene, the Fermi level is 0.08 0.16, 0.18, 0.35 and 0.55 eV respectively above the Dirac point for C, Si, Ge, Si-Ge co-decorated systems. In Fig. 5, we show the spatial distribution the transferred electrons i.e., the charge within the energy window from the Dirac point to the Fermi level, on the graphene surface. This partial charge density analysis shows that electron transfer from the underlying decorated patches is not distributed uniformly on graphene. More electrons are seen in decorated graphene areas than in the non-decorated regions, as can be seen from the partial charge distribution in Fig. 5.

**Figure 6:**
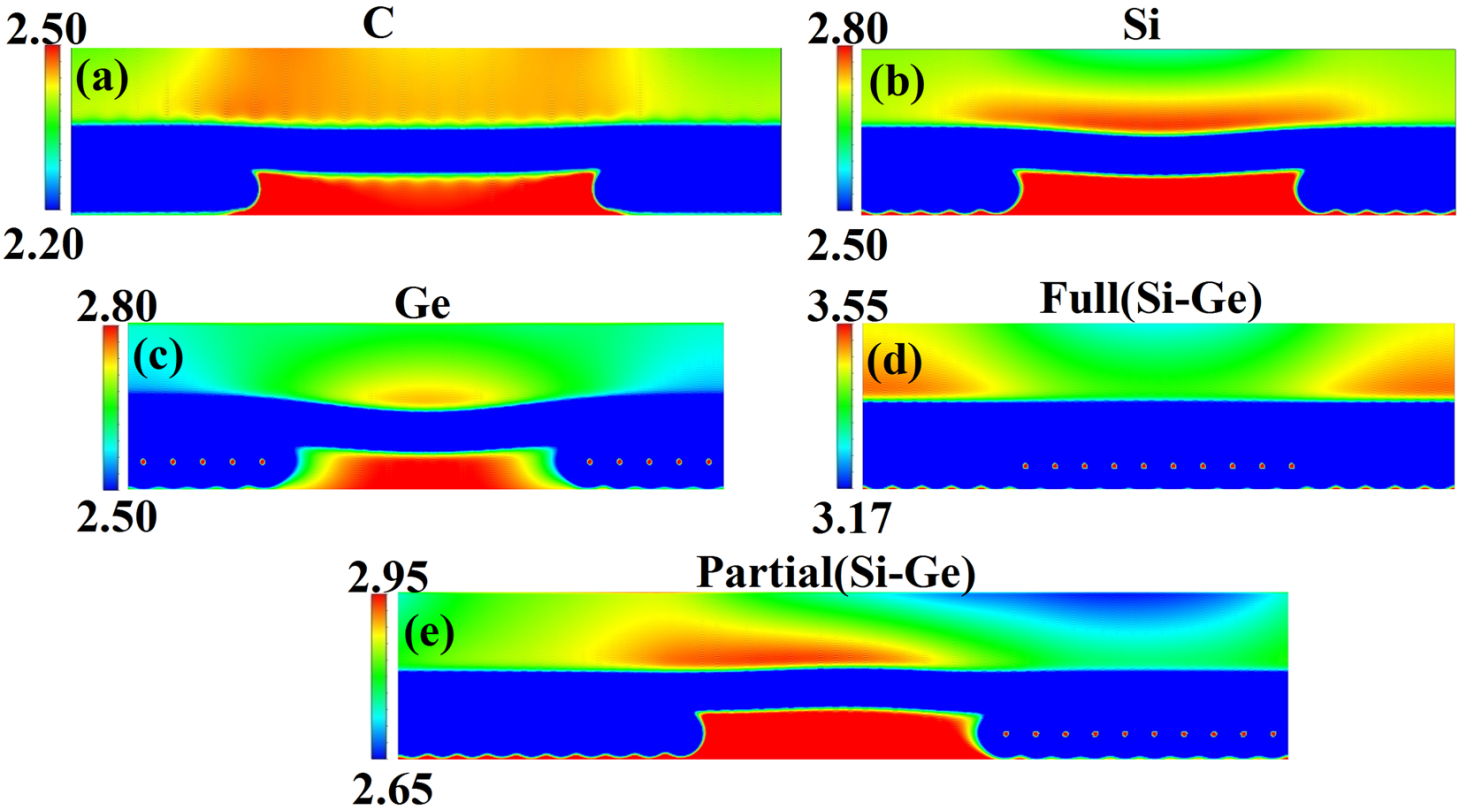
2D electrostatic potential (in V) of partially C (**a**), Si (**b**), Ge (**c**), fully Si-Ge (**d**), and partially Si-Ge (**e**) decorated graphene. The side views of the potentials as recorded on the vertical cutting plane through the middle of the unit cell used in the calculation. The non-decorated domain is in the middle of the structure in (**a**)~(**c**) and (**e**).

**Figure 7:**
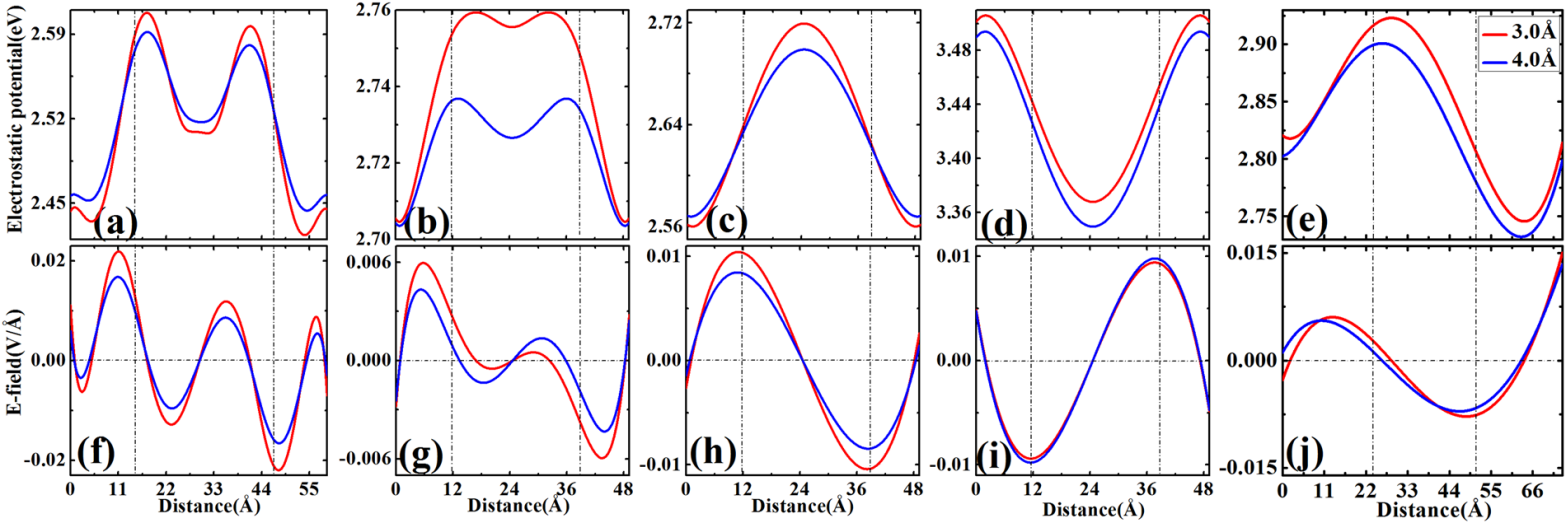
A line scan of the electrostatic potential and electric field from Fig. 6, respectively, at 3 and 4 Å from the graphene with partial C (**a**) and (**f**), Si (**b**) and (**g**), Ge (**c**) and (**h**), full Si-Ge (**d**) and (**i**), and partial Si-Ge (**e**) and (**j**) decoration. Note that the dash lines indicate the boundary of decorated and non-decorated domains.

Due to the non-uniform distribution of the transferred electrons, the strength of electrostatic potential are different at different location on graphene, as shown in Fig. 6. In order to get more insights into electrostatic potential distributions, we plot the electrostatic potential line scans at different heights of 3 and 4 Å above the graphene surface as shown in Fig. 7. It can be seen from Figs. 6 and 7 that, for partially C, Si, and Ge decorated graphene, the areas with underlying decorated C, Si, and Ge patches have lower electrostatic potential for the electrons while the potential in the non-decorated domain is approximately 0.06~0.2 eV higher as shown in Fig. 7(a)~(c). We have also calculated the electrostatic potential profiles for the systems with different lateral dimensions, i.e., 20 × 1 for Si, Ge and 24 × 1 for C. The results from 20 × 1 and 24 × 1 samples are very similar as one can see from Fig. 7, indicating that our calculation using 20 × 1 is converged.

In the case of fully Si-Ge co-decorated graphene one can see from Fig. 7(d) that the electrostatic potential exhibits larger value in the area with Si-decorated than that with Ge-decorated by about 0.17 eV. It can be also seen that the electrostatic potential is complicated in partially Si-Ge co-decorated graphene, as shown in Fig. 7(e). The electrostatic potential in Ge-decorated area is smaller than that in Si-decorated area, but the potential at both decorated areas are smaller than that of pristine areas. The local potential difference between decorated and non-decorated domains or between different elements decorated domains will cause a local work function difference between local domains, which can be attributed to the uneven electron distribution discussed above.

The electric field distribution induced by C, Si, Ge decorations or Si-Ge co-decoration can be evaluated by taking the gradient of the electrostatic potentials along the line scan direction. These results are shown in Fig. 7(f)~(j) respectively. An alternating electric field across the domains of different decorated and pristine graphene region is observed with the largest field (e.g., 0.12~0.37 × 10^6^ V/cm at height of 3 Å) at the domain noted that the distributions of electrostatic potentials in partially C and Si decorated graphene is more complicated than the other cases and exhibit a bump as shown in Fig. 7(a) and (b). However, the oscillatory behavior of the electric field still can be seen from Fig. 7(f) and (g). Such alternating electric fields can provide a significant driving force for the motion of adatoms or molecules on the partially decorated graphene if the boundaries indicated by vertical dash lines. It should be adatoms or molecules are charged^10,22^. We noted that using adsorption or decoration of other materials to modify the properties of graphene have been attracted a lot of current interest. While our present paper discusses the induced in-plane electric field and its influence on the diffusion and nucleation of adatoms on graphene, induced magnetic field has also been recently studied by adsorption of organic molecules on graphene surface^33^.

The current finding also indicates that the strength of the induced electric field can be manipulated by controlling the decorated patterns or decorated elements. For instance, our calculation results show that partial decoration with different elements, i.e., C, Si, Ge or their mixtures, can results in different electric field distributions. Therefore, through manipulating the decorated pattern and/or the type of decoration element, such induced electric filed mechanism can be utilized to control the adsorption sites, nucleation areas as well as growth morphology of metal on graphene. Recent experiments have shown that inhomogeneous graphene supported with mixed areas of different intercalations can be achieved by using Ge as the intercalant for graphene grown on SiC^18,19^, giving rise to a quasi-free standing graphene with two distinct areas: an n- and a p-doped graphene. It has also been shown that by controlling the interaction it is possible to generate three types of regions: pristine, n-doped, and p-doped areas that have potential use as in-plane transistors. We note that our present finding is very relevant, showing that the electron doping, electrostatic potential and induced electric field can be manipulated by controlling the decorated patterns or decorated elements. Such decorations not only can guide the charged atoms to the areas of stronger adsorption but also play an important role in nanostructure nucleation and growth on graphene^10,22^. We also noted that although our present decoration modeling is not the same as intercalation because the effects of the substrates are not considered in our calculation due to the heavy computational costs, the main observation and conclusions from the present study should be similar to the cases of intercalation, especially when the substrates are carefully chosen to minimize the interaction between the intercalated layers and the substrates.

## Conclusion

By using first-principle calculations we studied the structures and electronic properties of C, Si, Ge, and Si-Ge decorated graphene. We show that the interaction between graphene and the underlying decorated patches is of Van der Waals character. The underlying decorated patches act as agents for electron doping of graphene without disturbing the graphene electronic π-bands. The redistribution of the electrons makes the electrostatic potential lower in the decorated graphene areas, and thus induced an alternating electric field across the boundary between the decorated and non-decorated domains. We note that inhomogeneous decorated graphene with mixed areas of different decoration could be useful for graphene-based devices.

We show that the electric field on graphene can be manipulated by controlling the decoration. The electric field induced by partial decoration can cause biased diffusion of charge metal adatoms on graphene, which can in turn be used to control the morphology of the grown metal^10,22^. This capability can be used to modify the adsorption properties of graphene by controlling the spatial distribution of the decorated areas to meet various requirements relevant to the technological applications of graphene for electronic and spintronic devices, which depends on the controlled distribution of metals to specific substrate locations.
